# Efficient Drone Detection Using Temporal Anomalies and Small Spatio-Temporal Networks

**DOI:** 10.3390/s26010170

**Published:** 2025-12-26

**Authors:** Abhijit Mahalanobis, Amadou Tall

**Affiliations:** Department of Electrical and Computer Engineering, University of Arizona, Tucson, AZ 85719, USA; amadoutall@arizona.edu

**Keywords:** infrared drone detection, anomaly detection, TCRNet, TRX detector, spatio-temporal analysis

## Abstract

Detecting small drones in Infrared (IR) sequences poses significant challenges due to their low visibility, low resolution, and complex cluttered backgrounds. These factors often lead to high false alarm and missed detection rates. This paper frames drone detection as a spatio-temporal anomaly detection problem and proposes a remarkably lightweight pipeline solution (well-suited for edge applications), by employing a statistical temporal anomaly detector (known as the temporal Reed Xiaoli (TRX) algorithm), in parallel with a light-weight convolutional neural network known as the TCRNet. While the TRX detector is unsupervised, the TCRNet is trained to discriminate between drones and clutter using spatio-temporal patches (or chips). The confidence maps from both modules are additively fused to localize drones in video imagery. We compare our method, dubbed TRX-TCRnet, to other state-of-the-art drone detection techniques using the Detection of Aircraft Under Background (DAUB) dataset. Our approach achieves exceptional computational efficiency with only 0.17 GFLOPs with 0.83 M parameters, outperforming methods that require 145–795 times more computational resources. At the same time, the TRX–TCRNet achieves one of the highest detection accuracies (mAP_50_ of 97.40) while requiring orders of magnitude fewer computational resources than competing methods, demonstrating unprecedented efficiency–performance trade-offs for real-time applications. Experimental results, including ROC and PR curves, confirm the framework’s exceptional suitability for resource-constrained environments and embedded systems.

## 1. Introduction

Imaging Infrared (IR) sensors are widely used for security and surveillance applications. Because of the sensitivity and ability to operate during the day and at night, IR sensors are a suitable choice for detecting small drones in scenes with challenging backgrounds. With the advent of advanced thermal imaging cameras, it is now possible to obtain the most detailed infrared image of the scene. In general, “cooled” IR cameras offer the best performance and the best image quality, while “uncooled “cameras are less expensive. The choice of IR cameras also depends on the required thermal sensitivity, i.e., its ability to distinguish differences in temperature. Typically, cooled medium-wave (3–5 microns) IR sensors are more sensitive than uncooled sensors that work in the long-wave (8–14 microns) part of the IR spectrum. This is an important consideration for detecting faint objects at longer ranges amidst thermal background clutter. The resolution of the IR image is determined by both the sensors’ field of view, and the number of pixels captured by the camera. In general, a greater number of pixels gives better spatial resolution, provided the aperture of the optical system is also capable of resolving the details in the image. In turn, this translates into greater detection range for smaller objects. Therefore, cooled IR cameras with high spatial resolutions are the sensors of choice for detecting drones at long distances. Finally, panoramic images are required to monitor large fields of view. This can be achieved either by using an array of fixed imagers or by a single camera with a scanning system. The array of cameras provides continuous coverage of the monitored field of view but also increases the data-processing requirements. On the other hand, a scanning system has greater latency between successive “looks” or “revisits” to a particular part of the field of view. Therefore, the design for monitoring a wide-field-of-view surveillance system must balance the data-processing requirements and cost with the need for persistent surveillance for drone detection.

An example of a drone detection system designed on the basis of the above consideration is described in reference [1]. In this system, a high-resolution thermal camera continuously rotates at a high speed of up to 2 Hz while providing outstanding image quality with very high spatial resolution (e.g., up to 120 Mpixels). This makes it possible to generate a video stream of high-resolution thermal panoramic image at a rate of up to 2 Hz. Such a system ensures continuous real-time monitoring over the entire surveillance area, while the high spatial resolution and high sensitivity ensure high detection rates of small drones at long range.

Another key consideration are the computing resources needed to keep up with the high rate at which the sensor data needs to be processed. This is particularly important for drone detection systems that often need to be deployed and operate on mobile surveillance platforms at the “edge”. Therefore, to make the systems portable and efficient, it is necessary to manage the computational complexity of the algorithms. In this paper, our focus is on developing light-weight, low-computational-complexity drone detection methods for infrared imaging sensors whose performance is comparable to that of state-of-the-art algorithms that require much more computing power and memory storage.

Given that IR cameras can sense small drones at long distances, it is necessary to develop methods which rely on both temporal and spatial information. For such objects, temporal appearance changes over multiple frames provide richer information than static single-frame analysis. Considering this, we propose an algorithm for processing spatio-temporal volumes in parallel using a temporal variant of the Reed Xiaoli (TRX) anomaly detector, and a light-weight CNN known as the Target to Clutter Ratio network (TCRNet) [2]. While the original TRX detector was proposed for statistical anomaly detection in hyperspectral data, we repurpose it for detecting anomalous changes in pixel intensities over time. Specifically, the TRX detector is a constant false alarm rate (CFAR) algorithm that generates large per-pixel anomaly scores due to the movement of drones across the scene. On the other hand, the TCRNet is trained to discriminate between drones and clutter by learning the features that separate them in the spatio-temporal volume. For training the TCRNet, 3D spatio-temporal chips are extracted from the full frame videos, but during inference, the full volume is processed directly by the network

For a given input video, the TRX detector generates per-pixel anomaly scores for the full scene, while TCRNet produces a threat detection confidence map for the same. The TRX–TCRNet architecture normalizes and additively combines the output of each algorithm to correctly localize targets while attenuating potential false alarms. This approach also ensures real-time efficiency by avoiding sequential dependencies, such as proposal generation and searching over many anchor boxes. The dataset utilized contains LWIR infrared sequences with small drones, presenting greater challenges than others, like the CVPR anti-drone dataset or Small90, due to its emphasis on extremely low-visibility targets, vast search areas, and dynamic motion in cluttered scenes. Our framework prioritizes efficiency over resource-heavy deep learning models and achieves nearly state-of-the-art performance (97.40 mAP_50_) with only 0.83 M parameters and 0.17 GFLOPs. In comparison, other recently published methods require 8.7–17 times more parameters along with much higher FLOPs (such as 145 times more for ACM (24.66 G) [3], 769 times more for TRIDOS (130.72 G) [4], and 795 times more for DNANet (135.24 G) [5]. This dramatic trade-off between task performance and computational cost makes the TRX–TCRNet uniquely suitable for embedded systems, mobile platforms, and resource-constrained environments where traditional deep learning approaches are impractical.

By integrating temporal and spatial information with unprecedented efficiency, our method outperforms both deep single-frame detectors and handcrafted trackers. Ablation studies highlight the potential of adopting a local TRX detector with varying window sizes to further enhance detection accuracy. This computational advantage positions our approach as a preferable solution for practical deployment scenarios where both high accuracy and resource efficiency are critical requirements. Future phases will expand evaluation to broader datasets, underscoring the pipeline’s potential for small-object detection and other tiny-object video applications across diverse hardware platforms.

The rest of the paper is organized as follows. Section 2 is a review of other recent works on the topic of drone detection. Section 3 describes the algorithmic details of our proposed approach. The details of experiments and evaluation methodology are given in Section 4. Section 5 is a detailed discussion of the results and the different ablations studies that we conducted. Finally, the conclusion and directions for future research are given in Section 6.

## 2. Background Review

Drone detection using infrared imagery is a challenging problem due to low resolution, diurnal variations, background clutter and environmental degradations. To combat these challenges, drone detection algorithms often use video imagery to extract spatio-temporal motion cues that single-frame analysis fails to capture adequately. By incorporating temporal information across multiple frames, these methods exploit changes in position, velocity, and appearance to distinguish moving drones from static or cluttered backgrounds.

Deep learning has revolutionized the detection of small targets in infrared imagery by enabling end-to-end feature learning that often surpasses traditional statistical methods in accuracy, albeit frequently at the expense of increased computational demands. Contemporary approaches emphasize multi-scale fusion, attention mechanisms, and spatio-temporal integration to address the inherent challenges of detecting small, low-contrast drones in cluttered environments. Early advancements, such as the Asymmetric Contextual Modulation (ACM) network [3] introduced by Dai et al. in 2021, focused on cross-layer feature fusion tailored specifically for infrared small targets. By asymmetrically modulating contextual information, ACM enhances dim targets while suppressing background clutter, demonstrating robust performance on single-frame datasets through efficient background modeling.

Subsequent developments have incorporated dynamic modeling of image structures and attention-based refinements. For instance, Li et al.’s RISTD [6] (2022) leverages dynamic image structure evolution to disentangle targets from complex backgrounds, adaptively enhancing scarce target features across temporal and spatial dimensions to mitigate false alarms. Similarly, Zhu et al.’s SANet [7] (2023) integrates a spatial attention network with global average contrast learning, amplifying small target signals by emphasizing spatial relationships and contrast disparities, which proves particularly effective in low-SNR scenarios.

Attention-guided architectures have further advanced the field, as seen in Zhang et al.’s AGPCNet [8] (2023), which employs pyramid contexts for multi-scale semantic association, capturing both global and local features to enable robust detection amid complex clutter. Shape-aware innovations, exemplified by Zhang et al.’s ISNet [9] (2022), prioritize geometric properties of targets, incorporating specialized modules to distinguish genuine objects from similar clutter through focused feature extraction.

Dual-network and nested designs have also emerged to handle multi-level representations. Wu et al.’s UIUNet [10] (2022) combines dual U-Net architectures for comprehensive multi-scale and multi-level feature learning, fusing local and global contexts to accommodate varying target sizes. Sun et al.’s RDIAN [11] (2023) extends this by introducing receptive-field and direction-induced attention, dynamically expanding receptive fields to capture diverse target orientations and directional cues in noisy environments.

More recent dense nested attention mechanisms, such as those in Li et al.’s DNANet [5] (2023), facilitate repeated fusion and enhancement of contextual information [12,13,14,15,16,17] through intricate interactions, bolstering feature representation for small targets and achieving high accuracy in single-frame processing. Spatio-temporal extensions have pushed boundaries further; Cai et al.’s SSTNet [18] (2024) utilizes a sliced spatio-temporal network with cross-slice ConvLSTM to process video slices, capturing motion dynamics with reduced latency in multi-frame settings.

Finally, Duan et al.’s TRIDOS [4] (2024) represents a comprehensive triple-domain strategy, integrating spatio-temporal-frequency features via Fourier transforms, spatial encoding inspired by human vision, and temporal motion capture through differential learning. This holistic approach overcomes limitations of purely spatio-temporal methods, yielding state-of-the-art results on benchmarks like DAUB [19], ITSDT-15K [20], and IRDST [21], while striving for a balance between accuracy and real-time efficiency.

These deep learning paradigms collectively highlight a shift toward hybrid, efficiency-optimized designs that leverage advanced attention and multi-domain fusion to tackle the unique demands of infrared drone detection. To broaden the technological landscape of small-target detection beyond infrared small-target methods, several recent studies offer relevant insights. Fang et al. [21] introduced SEB-YOLOv8s, a real-time model emphasizing enhanced sensitivity to small aerial objects under constrained imaging conditions. Brighente et al. developed ADASS [22], an embedded audio-based anti-drone sentinel, showing the emerging importance of multimodal sensing. RF-based detection also remains active, as demonstrated by the distributed hardware-accelerated system of Flak and Czyba [23]. Recent advancements in airborne object recognition, such as the lightweight deep learning approach of Hlavata et al. [24], highlight cross-domain strategies applicable to UAV monitoring. General-purpose small-object detectors continue to evolve as well; for example, the Improved YOLOv10’s Real-Time Object Detection Approach in Complex Environments [25] and the Dist-Tracker framework—designed specifically for UAV tracking [26]—illustrate the growing trend toward integrating temporal cues and lightweight computation. These developments reinforce the motivation for our work: to design a resource-efficient spatio-temporal detector that remains competitive with recent deep architectures while maintaining a minimal computational footprint [27].

## 3. Proposed Model

**Figure 1:**
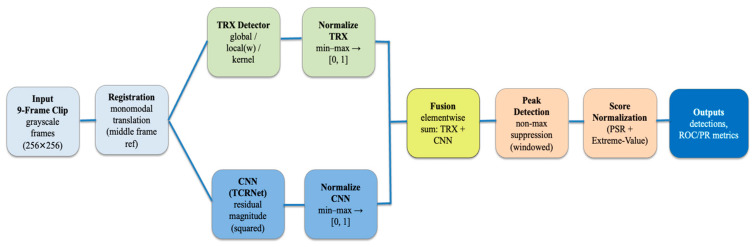
Overview of the proposed framework for the TRX–TCRNet model.

Deep learning methods for drone detection are computationally expensive and require large amounts of training data. To avoid these issues, we propose combining a statistical outlier detection method (i.e., the TRX anomaly detector) with a light-weight CNN (the TCRNet), as shown in Figure 1. Assuming a stationary background, the TRX anomaly detector treats the temporal variations that occur due to noise at every pixel as a gaussian random process. Drones moving across the scene cause the pixel values to deviate drastically, which are then easy to detect as statistical outliers. As the video frames are received, the TRX anomaly detector dynamically estimates the mean and covariance matrix at every pixel over the observation window and does not require any prior training. The TCRNet has been previously used for detecting stationary ground targets in single frame imagery. However, for our application, we generalize it to discriminate between clutter and small aerial targets using both temporal and spatial features. Specifically, the filters in the first layer of the TCRNet are analytically derived as the 3D eigenbasis for spatio-temporal features that best separate moving drones from stationary clutter. Holding the first layer fixed, the rest of the network optimizes the TCR metric to maximize detection while minimizing false positives. This parallel combination of the TRX detector and TCRNet ensures efficient computation, as both algorithms operate on the full spatio-temporal volume without sequential dependencies, minimizing latency while maintaining low overhead.

### 3.1. Temporal Extension of Reed Xiaoli Detector

While the original TRX detector is widely used for locating anomalies in hyperspectral data, we believe this is the first time it has been modified to find temporal anomalies in IR video streams for drone detection. Previous works which have used the TRX detector for temporal analysis are in the context of satellite image analysis and for finding dim moving objects in hyper-spectral data [28]. Consider a stack of N successive image frames obtained from a video. At each pixel, we define a temporal observation vector given by $v_{i}={\left[\;v_{i1}\;\;v_{i2}\;\;\ldots \;\;v_{iN}\;\right]}^{T}$, where $v_{ij}$ represents the value of the *i-*th pixel in the *j-*th frame. The TRX detector estimates the mean and covariance matrix of all such vectors that lie inside a sliding “double” window. This window encompasses the pixels that lie inside a larger outer window but outside a smaller inner window centered at the pixel in the middle. Thus, for the double window centered on the *k-*th pixel (and encompassing a total of *M* pixels), the mean is estimated as $\mu_{k}=\frac{1}{M}\sum_{i\neq k}v_{i}$, and the covariance matrix is $C_{k}=\frac{1}{M}\sum_{i\neq k}\left(v_{i}-\mu_{k}\right){\left(v_{i}-\mu_{k}\right)}^{T}$. The anomaly score at the *k-*th pixel is then given by

(1) $${rx}_{k}=\left(v_{k}-\mu_{k}\right)C_{k}^{-1}{\left(v_{k}-\mu_{k}\right)}^{T}$$

which is the Mahalanobis distance between the mean vector of the pixels contained inside the window, and the test pixel at its center. This quantity is computed at all pixel locations across the scene to form the output anomaly score map.

### 3.2. Review of TCRNet

**Figure 2:**
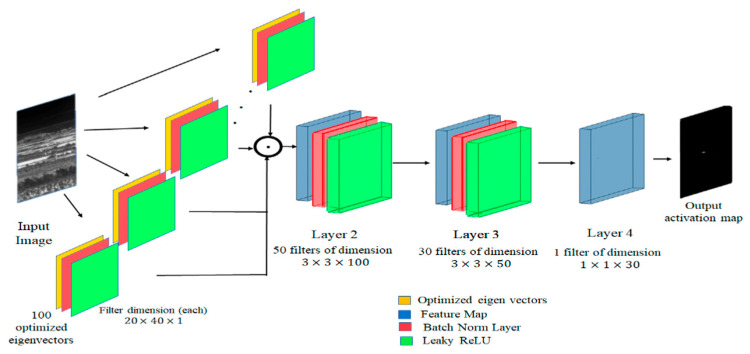
The architecture of the TCR network. Layer 1 is analytically derived, while the rest of the convolutional layers are iteratively learned to optimize the TCR metric.

The TCRNet (Figure 2) is a compact CNN designed for efficient target detection in the cluttered environments. The original TCRNet was trained to detect ground targets in infrared image frames, by maximizing the TCR metric [2,29]. Conventional regression-based training methods minimize the Mean Squared Error (MSE) loss between the actual and ideal desired response of the network. However, we observed that this approach does not work well for our application where the shape of the desired response is not important. Rather, it is essential to produce a strong response at the true location of the targets, while attenuating the output of the network produced in response to clutter. We now provide a brief description of the TCR metric and how it is used as a cost function for training the network.

The TCRNet is unique in that the filters in the first layer are obtained by analytically maximizing the TCR metric [2]. This was found to be helpful for training with smaller datasets and fewer training images. Holding these filters fixed imposes strong priors for rest of the network which can be trained in the usual manner to optimize the TCR metric. Assume that w represents the filter, while $C_{x}$ and $C_{y}\;$represent the covariance matrices of the target and clutter training chips. It can be shown that the average squared magnitude of filter’s output in response to targets is given by $w^{T}C_{x}w$. Similarly, the average squared magnitude of the same filter’s response to clutter is given by $w^{T}C_{y}w$. The filters in the first layer are obtained by maximizing the ratio of these two quantities, i.e.,

(2) $$TCR=\frac{w^{T}C_{x}w}{w^{T}C_{y}w}$$

This is the well-known Raleigh quotient, which is maximized by choosing w to be the eigenvectors of $C_{x}^{-1}C_{y}$. The eigenvectors with larger eigenvalues favor targets while the ones with smaller eigenvalues favor clutter. Thus, the dominant eigenvectors are chosen to be the filters in the first layer of the TCRNet.

While the filters in the first layer are held fixed, the rest of the network is trained as follows. Let us assume that we have $N_{x}$ and $N_{y}$ labeled samples for the target and clutter classes, and their responses at the output of the network are denoted by the vectors $\begin{array}{cccc} x_{1} & x_{2} & \ldots & x_{N_{x}} \end{array}$ and $\begin{array}{cccc} y_{1} & y_{2} & \ldots & y_{N_{y}} \end{array}$ respectively. The L2-norm of these vectors, i.e., $x_{i}^{T}x_{i}$ and $y_{i}^{T}y_{i}$, is referred to as the output energy produced in response to target and clutter samples, respectively. During training, the response to targets (i.e., $x_{i}$) can be obtained from the network’s output using the ground-truth information. It can represent either the output score at the location of the centroid of the target, or a vector of scores at all locations within the ground-truth bounding box. Similarly, $y_{i}$ is a vector of output scores obtained when a clutter sample is presented at the input. Our objective is to maximize the energy in the output when targets are present and minimize the same in response to clutter. This is accomplished by minimizing

(3) $${\overset{´}{J}}_{TCR}=\frac{\frac{1}{N_{y}}\sum_{i}y_{i}^{T}y_{i}}{\frac{1}{N_{x}}\sqrt{\prod_{i}x_{i}^{T}x_{i}}}$$

which is the ratio of the arithmetic mean of the energy of the clutter outputs to the geometric mean of the energy of the target outputs. Minimizing this ratio will make the numerator of ${\overset{´}{J}}_{TCR}$ small, which in turn ensures that all the terms in the summation $\frac{1}{N_{y}}\sum_{i}y_{i}^{T}y_{i}$ are small. Similarly, the denominator of ${\overset{´}{J}}_{TCR}$ must be large to minimize the ratio, which implies that $\frac{1}{N_{x}}\sqrt{\prod_{i}x_{i}^{T}x_{i}}$ is large, and that in turn ensures that each term in the product is also large. It can be shown that the derivative of the log of ${\overset{´}{J}}_{TCR}$ with respect to each class is

(4) $$\nabla_{y_{i}}log\left({\overset{´}{J}}_{TCR}\right)=\frac{2y_{i}}{\sum_{i}y_{i}^{T}y_{i}}$$

for clutter samples, and

(5) $$\nabla_{x_{i}}log\left({\overset{´}{J}}_{TCR}\right)=\frac{-1}{N_{x}}\frac{2x_{i}}{x_{i}^{T}x_{i}}$$

for target samples.

Therefore, as training images are presented to the network during the learning process, the gradient supplied to the back-propagation algorithm is either $\nabla_{y_{i}}log\left({\overset{´}{J}}_{TCR}\right)$ for clutter samples, or $\nabla_{x_{i}}log\left({\overset{´}{J}}_{TCR}\right)$ for target samples. It should be noted that for one training image at a time, the gradient expression for the two classes reduces to $\nabla_{y_{i}}log\left({\overset{´}{J}}_{TCR}\right)=\frac{2y_{i}}{y_{i}^{T}y_{i}}$ and $\nabla_{x_{i}}log\left({\overset{´}{J}}_{TCR}\right)=\frac{-2x_{i}}{x_{i}^{T}x_{i}}$, which are simply the energy-normalized outputs produced by the training samples.

### 3.3. Output Fusion and Peak Detection Strategy

The outputs of both the TRX detector and the TCRNet are the same spatial size as the input image. This makes it straightforward to additively combine them after adjusting their dynamic range. The TRX output is always positive since it is a squared distance measure. To ensure that the TCRNet output is also positive, we take its squared magnitude. Both outputs are normalized to values between 0 and 1.0, and then directly added. Peaks are detected by searching for local maxima in the final output, and their numerical values are recorded along with their row and column positions. All other values within a small window centered at the peak location are discarded.

Even though the peak values are normalized, they do not represent a confidence probability. It should be noted that peak values, by definition, occur on the tail of the distribution of values present in the output. It is also well-known that the behavior of samples from the tail of any distribution is governed by the Extreme Value (EV) distribution. Therefore, to convert the detection values (say *x*) to probability measures, we fit them to the Gumbel distribution given by

$$f\left(z\right)=\frac{1}{\beta }e^{-(z+e^{-z})}$$

where $z=\frac{x-\mu }{\beta }$, and μ and β are knowns the location and scale, respectively. Furthermore, the mean is given by $E\left(x\right)=\mu +\gamma \beta$ while the standard deviation is $\sigma =\pi \beta /\sqrt{6}$. Here, γ is the Euler–Macheroni constant and equals 0.5772. The process for mapping the raw scores to the EV distribution is as follows. Given a list of *N* detection scores $\left\{x_{i}\right\},1\leq i\leq N$ we estimate E(x) as the sample average $\bar{x}=\frac{1}{N}\sum_{i}x_{i}$, and the standard deviation as $\sigma_{x}=\sum_{i}{\left(x-\bar{x}\right)}^{2}$. Given these two quantities, the parameters of the Gumbel distribution can be estimated as

$$\beta ={\sqrt{6}\sigma }_{x}/\pi ,$$

$$\mu =\bar{x}-\gamma \beta$$

Thereafter, the raw detection scores are standardized using $z_{i}=\frac{x_{i}-\mu }{\beta }$, 1≤i≤N.

In practice, the parameters μ and β are computed for every output frame, so that the detection scores for every output frame are fitted to the EV distribution. This ensures that the detection scores are comparable across all frames and data sequences.

## 4. Methodology and Experiments

**Figure 3:**
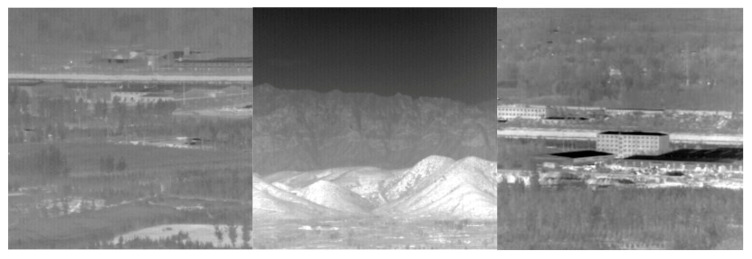
Example images of input data used for training and testing.

We evaluated the proposed model on the Detection of Aircraft Under Background (DAUB) dataset [19]. Sample frames of the dataset are shown in Figure 3, and Table 1 shows the specifications of the dataset. This dataset comprises real mid-wave infrared video sequences captured using a cooled 3–5 µm camera with 256 × 256 resolution, 3.0° × 3.0° FOV, and up to 100 Hz frame rate. It includes 22 data segments, 30 trajectories, and 16,177 frames containing 16,944 annotated targets. The UAVs are small fixed-wing aircraft appearing as 1–10-pixel targets at low altitude. The dataset spans diverse conditions—day/night cycles, sunny and cloudy weather, and backgrounds including sky, vegetation, suburban areas, terrain, and man-made structures—providing a challenging benchmark for dim-small target detection.

The training set consists of ten video sequences with a total of 8983 frames, while the test set contains seven videos with 4795 frames. For fair comparisons with other detection methods, following [4,18], we gauge performance using the Precision and Recall (PR) curve metric, the F1 score, and the mean average precision (mAP) with an IoU threshold of 0.5. In typical drone detection scenarios, the number of true target pixels is significantly smaller compared to the vast background regions, creating a highly imbalanced distribution where negative samples (background) vastly outnumber positive samples (targets). Under such conditions, the PR curve provides a more informative assessment of model performance than traditional ROC curves, as it directly focuses on the positive class performance and is more sensitive to changes in the number of false positives when the negative class dominates the dataset. The PR curve effectively captures how well the model maintains precision as recall increases, which is essential for evaluating detection systems where minimizing false alarms while maximizing target detection is critical. Specifically, the evaluation metrics are defined as follows:

(6) $$Precision=\frac{TP}{TP+FP}$$

(7) $$Recall=\frac{TP}{TP+FN}$$

(8) $$F1=\frac{2\times Precision\times Recall}{Precision+Recall}$$

where TP, FN, and FP represent the number of true positives (correct detections), false negatives (missed targets), and false positives (false alarms), respectively. The F1 measure is a useful metric that combines both Precision and Recall.

We also present the results in the form of the Receiver Operating Characteristic (ROC) curve to compare different variations in our approach. We define probability of detection (Pd) as the ratio of the number of true targets detected to the total number of true targets in the test data. The false alarm rate (FAR) is defined as

(9) $$FAR=\frac{Total\;number\;of\;false\;positives}{Total\;number\;of\;frames\times FOV}$$

where FOV is the product of the horizontal and vertical fields of view of the sensor. The ROC and PR curves are shown for the entire test set.

The Area Under the Curve (AUC), which provides a quantitative analysis of the model, is the area under the ROC or PR curve. In practice, AUC is often used as an index to evaluate the method’s accuracy. The larger the AUC value, the better the detection performance of the algorithm.

### Experiment Settings

**Figure 4:**
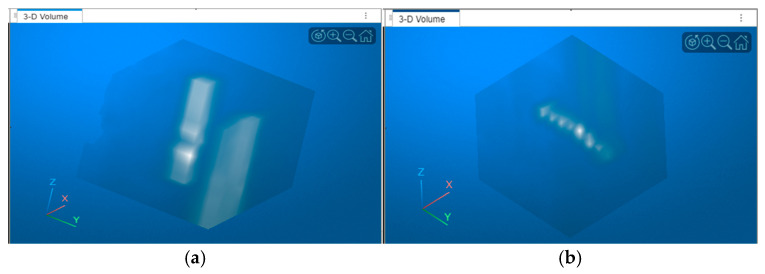
Volumetric images showing 11 × 11 × 9 training cubes of (**a**) clutter and (**b**) a moving target.

Input videos are partitioned into non-overlapping blocks of 9 consecutive frames to capture temporal motion cues of small drones. Within each block, frames are registered to the central frame using translation alignment, thereby compensating for effects of camera motion or shifts. Dead pixels are corrected by replacing them with the frame’s mean intensity. The registered block forms a spatio-temporal volume (STV) of size, where the first two dimensions are the height and width of the image frames, and the third dimension represents the temporal depth (Figure 4). The TCRNet is trained using “cubes” (or smaller STVs) of size 11 × 11 × 9 extracted from the training videos using ground-truth locations for the drones. Clutter training samples are also extracted from this data at the locations of the false alarms produced by the TRX detector. These training cubes are used to compute the 3D basis filters for the first layer of the TCRNet and for training the rest of the network. During inference on the test set, the full STV is processed in parallel by the TCRNet and the TRX detector as shown in Figure 1.

The architecture of the TCRNet is as follows:

- Image input layer for 256 × 256 × 9 volumes.
- Fixed 2D convolution (11 × 11 × 9 kernel, 300 filters) using the precomputed basis set ∅, with learning disabled to retain statistical priors.
- ReLU activation.
- Learnable convolutions: 3 × 3 × 128 with batch normalization and ReLU, followed by 1 × 1 × 1 with batch normalization.
- Final 1 × 1 × 1 convolution producing a single-channel 256 × 256 confidence map.

The padding option is set to ’Same’ to preserve dimensions, and the network reconstructs a map with peaks at target centers. Training employs RMSProp (initial LR $10^{-5}$, L2 regularization 0.01, decay 0.65 every 50 epochs, 500 epochs, mini-batch 64). Ground-truth outputs have a center peak for targets and zeros for clutter. The network has 0.83 M parameters, and requires 0.17 GFLOPs (dominating total compute, as TRX requires 0.001 GFLOPs for 256 × 256 × 9).

After the score maps of the TCRNet and the TRX detector are normalized (between 0 and 1) and added, any edge artifacts are masked (with a 10-pixel border zeroed), and up to 20 peaks are detected using non-max suppression. It should be noted that the number of peaks to be detected is a user-selected parameter and not a fixed setting. We verified that the detector’s qualitative behavior is stable with respect to this parameter: halving K (e.g., K = 10) or doubling it (e.g., K = 40) yields ROC and PR curves with nearly identical shapes and ordering. Differences appear only in the extreme low-precision regime where very large false-alarm allowances are evaluated. Thus, K = 20 serves as a practical cap that prevents unbounded false positives without biasing comparative performance. The detection scores (peak values) are transformed to fit the EV distribution (see Section 5.2). Furthermore, detections within 5 pixels of ground truth are treated as true positives while all others are false alarms. Ablation studies are performed to evaluate local TRX window sizes (e.g., 11, 21, 35) for robustness.

All experiments were conducted on an NVIDIA GeForce RTX 4070 GPU with 12 GB VRAM. The computational framework utilized Python 3.9 and PyTorch 2.1.1, accelerated by CUDA 12.1. Hyperparameters were configured as follows: input image resolution of 256 × 256 pixels, RMSprop optimizer with initial learning rate 1 × 10^−5^, and weight decay 0.01 for regularization. Training employed a batch size of 64 to balance computational efficiency and model performance. The model underwent 500 training epochs to ensure convergence.

## 5. Discussion of Results

In this section, we discuss the performance of the TRX–TCRNet model and compare it against various other models (cited as references), while emphasizing its efficiency and accuracy. All networks are trained as described in their respective references. Detection is treated as a two-class problem, where a prediction is considered a true positive if its location is within five pixels of the ground-truth center (regardless of classification output). Otherwise, all other detections are treated as false positives.

**Figure 5:**
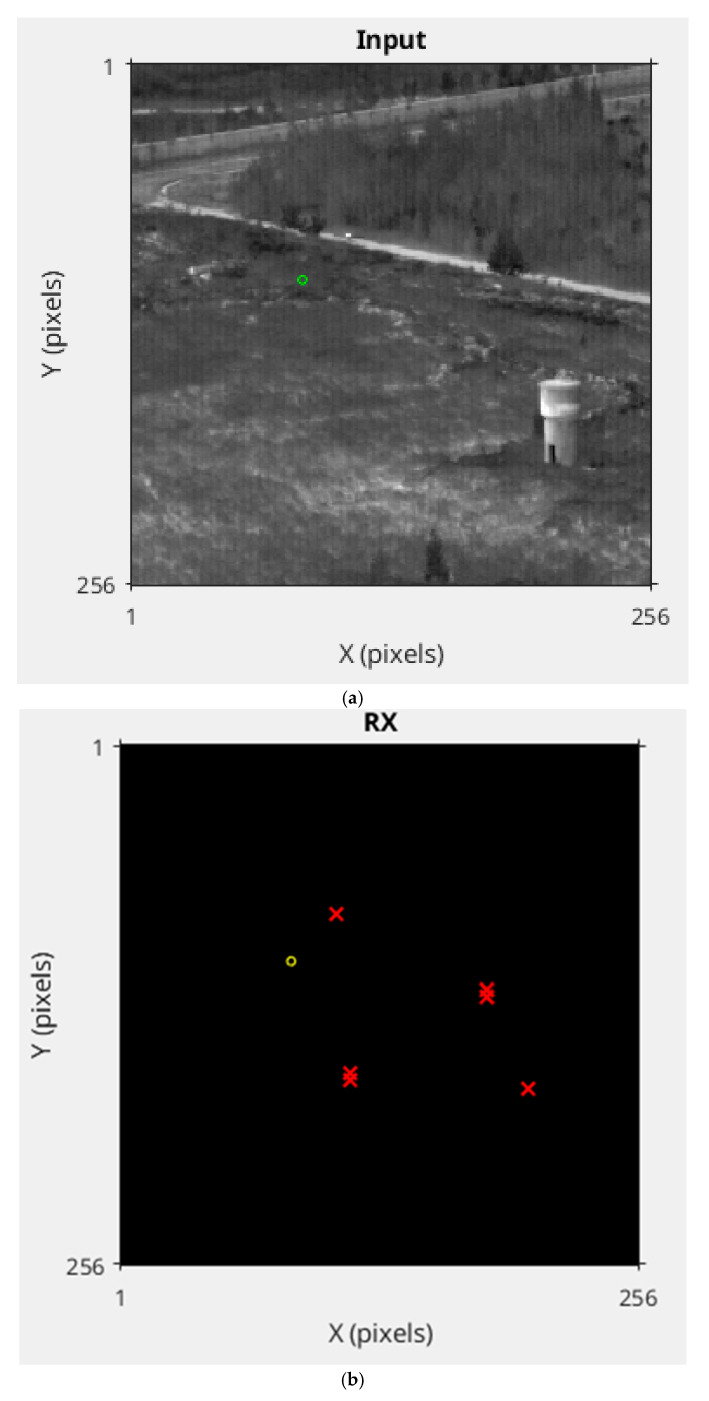

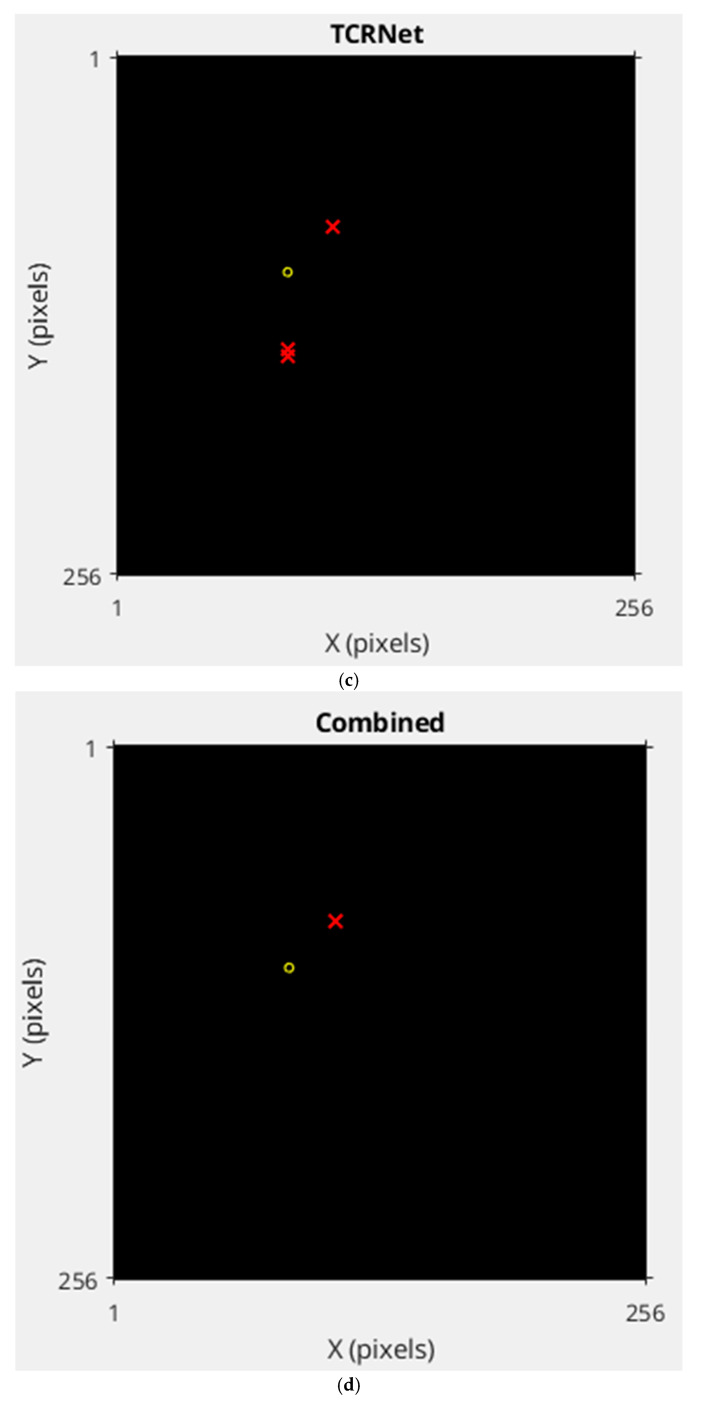
An illustration of the intermediate and fused outputs of the TRX–TCRNet pipeline. Panel (**a**) shows the original input frame where the target is indicated by a small green circle. Panel (**b**) displays the TRX anomaly map, where the true target (small circle) and false positives (red “x’) caused by background clutter are visible. Panel (**c**) shows the TCRNet confidence map, which correctly highlights the target but also produces several false alarms due to hard negatives. Panel (**d**) shows the fused map after additive combination and normalization, where the true target response is enhanced and most false positives are suppressed.

The TRX–TCRNet framework leverages the TRX detector’s statistical robustness to highlight anomalies and TCRNet’s learned spatio-temporal features to suppress clutter, achieving high accuracy with minimal computational overhead. Figure 5 illustrates example outputs from the pipeline, demonstrating the parallelism and fusion process. Specifically, the input image (a) is processed independently and in parallel by the TRX detector and TCRNet, producing anomaly maps (b) and confidence maps (c), respectively. In this example, the TRX output in (b) identifies the correct targets as a temporal anomaly along with three false alarms due to low SNR and background clutter. Similarly, the TCRNet output in (c) detects the correct target using spatio-temporal features while also finding three false alarms due to hard negatives like environmental artifacts. These parallel outputs are then normalized and additively fused to create the combined map (d). This enhances the true target peak, while attenuating the false alarm scores and thereby reducing the number of false alarms to two. This fusion exploits the strengths of both modules: TRX’s unsupervised sensitivity to motion outliers complements TCRNet’s supervised clutter rejection, resulting in improved localization and fewer spurious detections overall.

**Figure 6:**
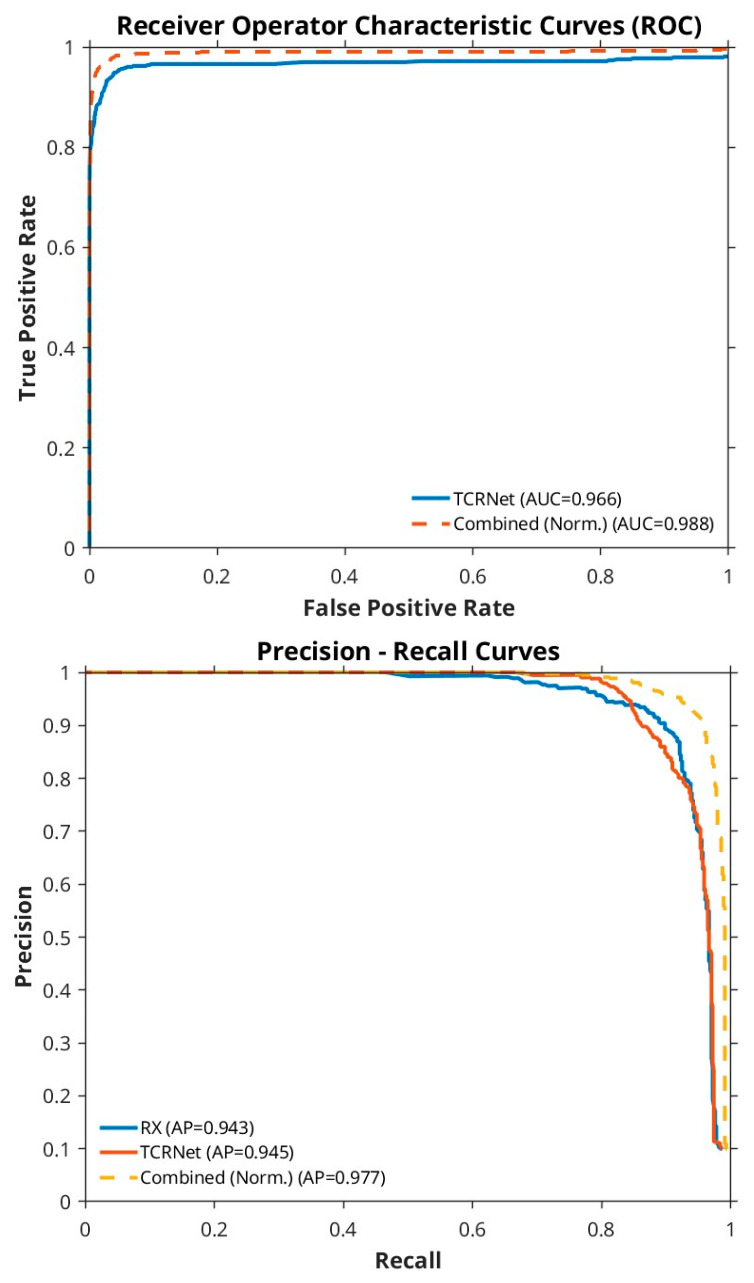
ROC and PR curves showing the performance of the model.

The ROC and PR curves in Figure 6 compare the performance contributions of the TRX detector (blue curve), the TCRNet (red curve) and the combined TRX–TCRNet framework (dashed yellow curve). The dashed yellow curve consistently envelopes the other two, highlighting the improvement gained by using these algorithms in parallel.

### 5.1. Comparison with Other Detection Methods

**Figure 7:**
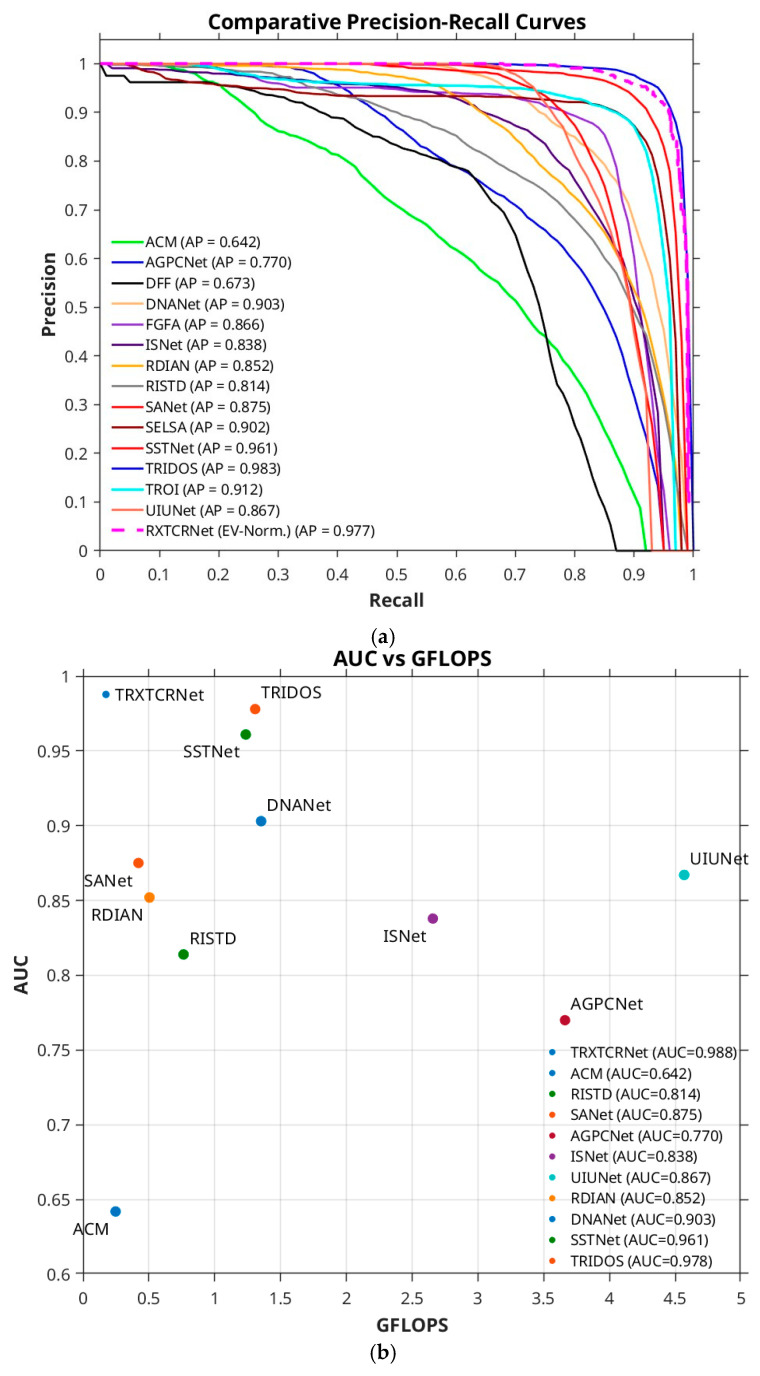
(**a**) PR curves of 15 representative detection methods, (**b**) scatter plot of the AUC of each model versus its FLOPs.

To compare the performance of TRX–TCRNet to that of other state-of-the-art methods, we plotted the PR curves of 15 representative methods, as shown in Figure 7a. TRX–TCRNet’s curve (dashed magenta line) is nearly comparable to that of TRIDOS, i.e., the best-performing model (blue curve), and has a larger curve envelope indicating superior performance. Figure 7b illustrates the comparison of different methods in terms of both performance and computational complexity by plotting their AUC versus computational cost (GFLOPs). We note that the TRX–TCRNet achieves one of the highest performances for the lowest computational cost, thereby making it an attractive choice for use in edge operations and in systems with limited computing and power resources [30].

To compare the performance of TRX–TCRNet with other state-of-the-art methods, we report in Table 2 both the accuracy and complexity metrics. Specifically, for each method the table lists the number of frames processed, detection accuracy (mAP_50_ and F1 score), computational cost (FLOPs), model size (parameters), and overall performance (AUC). Importantly, the comparison spans both single-frame algorithms (e.g., ACM, DNANet) and multi-frame approaches (e.g., SSTNet, TRIDOS, TRX–TCRNet), thereby emphasizing how temporal integration can improve detection while also introducing computational trade-offs. The following observations highlight TRX–TCRNet’s strengths.

- Performance Advantages

TRX–TCRNet achieves an exceptional mAP_50_ of 97.40, closely trailing TRIDOS (97.80) by only 0.40 points, outperforming all single-frame methods. The next best single-frame approach, DNANet, achieves 89.93 mAP_50_, a 7.47-point gap, underscoring TRX–TCRNet’s superior detection capability in multi-frame settings.

- Computational Efficiency Excellence

TRX–TCRNet’s standout feature is its computational efficiency, requiring only 0.17 G FLOPs and 0.83 M parameters—the lowest among all compared methods. In contrast, TRIDOS demands 130.72 G FLOPs (769 times more) and 14.13 M parameters (17 times more), while DNANet requires 135.24 G FLOPs (795 times more) and 7.22 M parameters (8.7 times more). This efficiency makes TRX–TCRNet ideal for resource-constrained environments and real-time applications.

- Trade-offs and Limitations

While TRX–TCRNet excels in efficiency, its F1 score of 92.50, though competitive, is lower than TRIDOS (99.43) and SSTNet (98.09), indicating a slightly weaker precision-recall balance. The lower F1 score of TCRNet stems primarily from the behavior of the TRX anomaly detector. TRX assigns elevated anomaly scores to many clutter regions due to deviations from its statistical background model, creating denser activation patterns in complex scenes. Although TCRNet produces stable chip-level classifications, the additive fusion can amplify moderate TRX responses in cluttered areas, slightly reducing precision and therefore F1 score. By contrast, TRIDOS and SSTNet employ deeply supervised spatio-temporal networks explicitly optimized for false-alarm suppression on DAUB. This illustrates a fundamental trade-off: our hybrid design provides strong efficiency and interpretability, while fully learned architectures achieve stronger precision through dataset-specific optimization. Additionally, processing nine input frames may introduce minor latency in real-time scenarios, though the low computational overhead mitigates this concern.

- Methodological Considerations

The parallel fusion of TRX and TCRNet confidence maps enables TRX–TCRNet to balance single-frame efficiency with multi-frame accuracy. Unlike methods like TRIDOS and DNANet, which achieve marginally better accuracy at significant computational cost, TRX–TCRNet’s design optimizes feature extraction and fusion for efficiency, processing full STVs without proposal generation overhead.

- Practical Implications

TRX–TCRNet offers an optimal solution for applications requiring high detection accuracy with limited computational resources, such as embedded systems and mobile platforms. Its ability to achieve near state-of-the-art performance with unprecedented efficiency bridges the gap between single-frame and multi-frame methods, making it highly suitable for real-time infrared drone detection.

### 5.2. Ablation Studies

To rigorously evaluate the contributions of key components in the TRX–TCRNet pipeline, we conducted ablation studies on two critical design elements: (i) extreme-value (EV) normalization of fused peak scores, and (ii) the operating mode of the Reed-Xiaoli (TRX) anomaly detector (global vs. local, with varying local window sizes). Unless otherwise specified, the TCRNet architecture, training protocol (RMSprop, 500 epochs, batch size 64), fusion strategy (additive combination of range-normalized TRX and TCRNet), non-maximum suppression (up to 20 peaks), and evaluation metrics (true positives within five pixels of ground-truth centers, PR/ROC curves, AUC, and AP) were held constant. EV normalization employs a per-frame Gumbel distribution fit, as described in Section 3.3, to calibrate raw detection scores into comparable probability-like measures across sequences.

**Figure 8:**
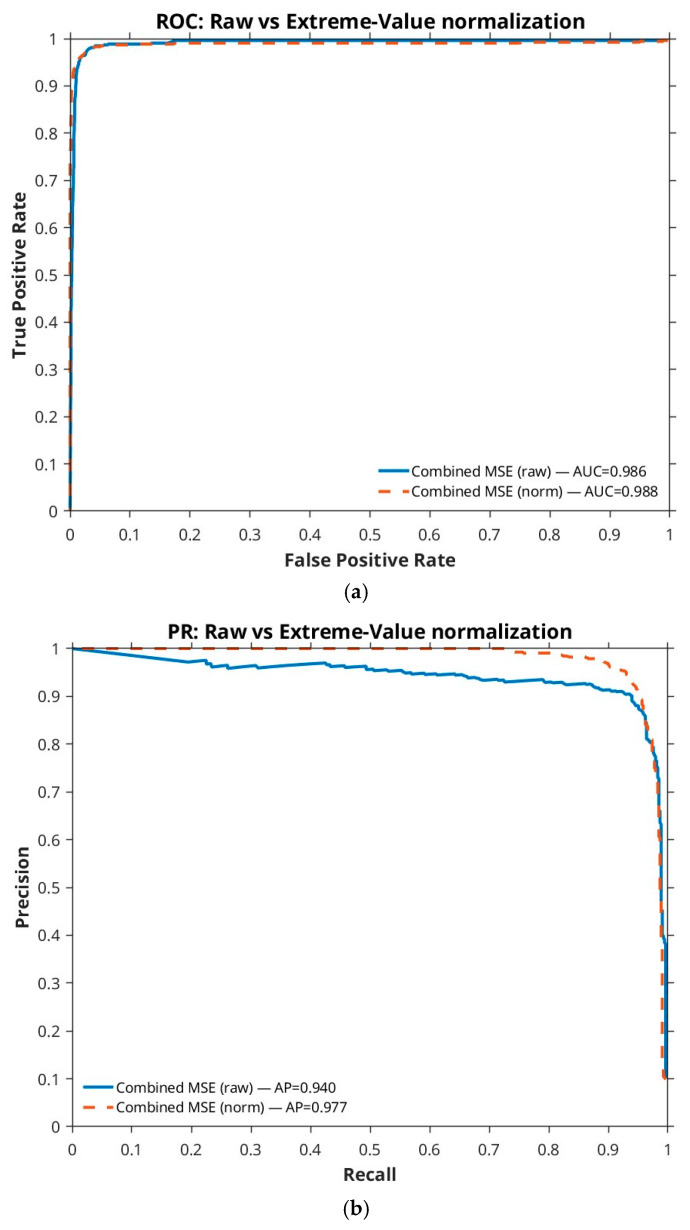
Effect of EV normalization on detection performance. (**a**) ROC curves comparing raw versus EV-normalized scores. (**b**) PR curves comparing raw versus EV-normalized scores.

Figure 8 illustrates the effect of EV normalization on detection scores. In panel (a), the ROC curves show that the EV-normalized variant (dashed orange curve) rises more steeply than the raw score baseline (blue curve). Similarly, in panel (b), the PR curve of the normalized method (dashed curve) remains consistently above the raw baseline (blue curve), indicating better calibration and precision at higher recall levels. EV normalization significantly enhances score calibration, addressing variability in raw score distributions caused by scene-specific clutter and differences in signal-to-noise ratio (SNR). Without normalization, raw scores exhibit unstable dynamic ranges, leading to inconsistent threshold performance across DAUB test sequences. By fitting scores to a Gumbel distribution, EV normalization compresses tail-end variability, yielding the following: (i) a 5–10% increase in precision at recall levels above 0.7 (Figure 8b), (ii) smoother PR curves with reduced oscillations (indicating stable operating points), and (iii) a 3–5% improvement in PR-AP and ROC-AUC. These gains, visible in Figure 8, stem from fewer false positives in low-contrast scenes (e.g., forest backgrounds) and more robust threshold transferability, making EV normalization critical for operational reliability in diverse environments.

**Figure 9:**
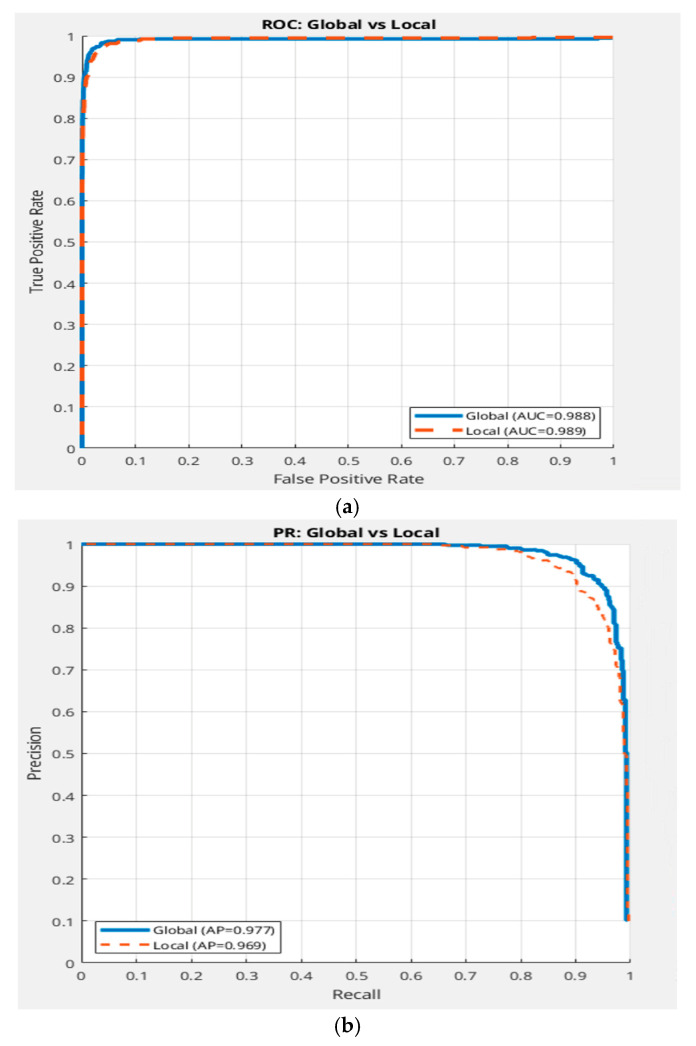
Global vs. local TRX performance. (**a**) ROC curves for global TRX versus local TRX (ω =35). (**b**) PR curves for global TRX versus local TRX (ω =35).

Comparing global TRX [31] (using full-frame background statistics) to local TRX (with a fixed window size ω = 35) reveals that global TRX [31] (blue curve) consistently outperforms its local counterpart, with a 4–6% higher PR-AP and a steeper ROC curve (Figure 9a,b). This counterintuitive result arises because local TRX with ω = 35 struggles with biased background estimates in scenes with large-scale, low-frequency clutter (e.g., suburban or plain backgrounds in DAUB). Target leakage—where drone pixels contaminate the local window—further degrades anomaly scores, increasing false positives by 10–15% compared to global TRX [31]. Global TRX, leveraging broader contextual statistics, better captures scene stationarity, making it more effective for the diverse clutter patterns in the DAUB dataset. This finding suggests that local TRX requires careful window size tuning to match the clutter scale.

**Figure 10:**
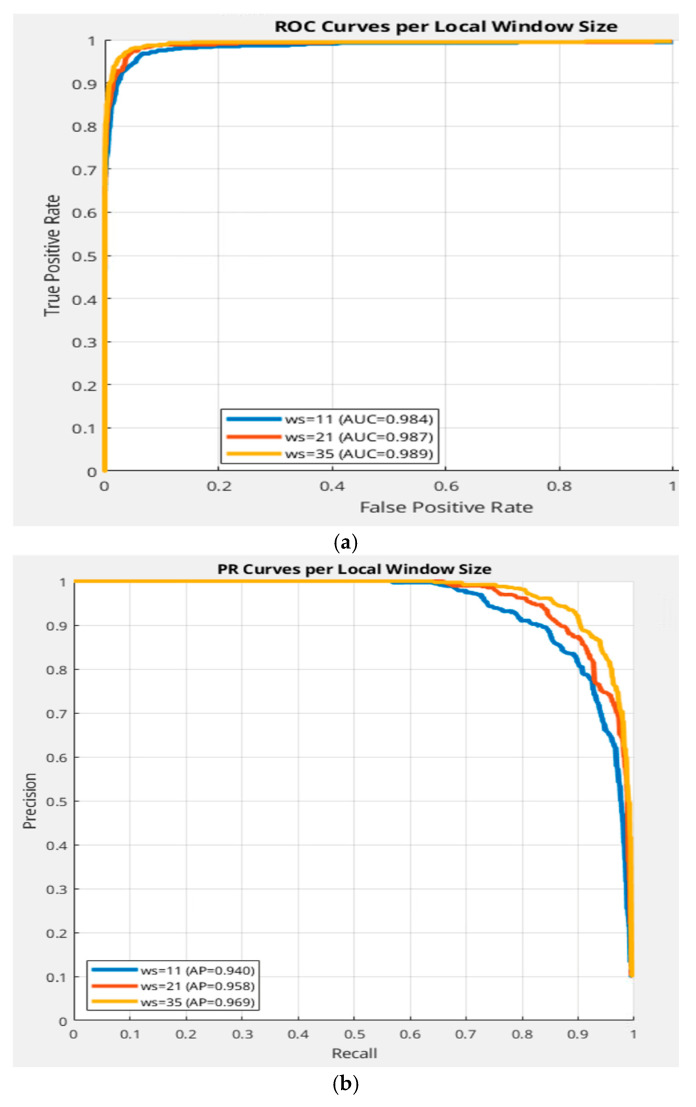
Effect of local TRX window size. (**a**) ROC curves for local TRX $\omega =\left\{11,21,35\right\}$. (**b**) PR curves for local TRX with $\omega =\left\{11,21,35\right\}$.

To assess local TRX performance, we evaluated the pipeline with window sizes ω=11,21,35. A clear monotonic trend emerges: larger windows improve detection performance, with ω =35 (yellow curve), achieving a 7–10% higher PR-AUC and 5–8% higher ROC-AUC compared to ω =11 (Figure 10a,b). Larger windows reduce estimation variance and mitigate target leakage, aligning local TRX performance closer to global TRX as ω increases. For instance, ω =35 yields a precision improvement of 8% at a recall of 0.8 compared to ω =11, reflecting better adaptation to spatially extended clutter. However, even at ω =35, local TRX falls short of global TRX by 2–3% in AUC, suggesting that further increases in ω or adaptive window sizing could bridge this gap. These results underscore the trade-off between computational cost and contextual scale, with implications for deploying TRX–TCRNet on platforms with varying computational constraints.

The ablation studies highlight EV normalization as a cornerstone of robust performance, ensuring consistent detection across diverse scenes. The superiority of global TRX in its current configuration highlights the need for adaptive or hybrid TRX strategies to strike a balance between local adaptability and global stability, particularly for real-time applications on embedded systems. Future work could explore dynamic window sizing based on scene complexity or integrate multi-scale TRX to further enhance performance without sacrificing efficiency.

### 5.3. Evaluation on the Anti-UAV410 Dataset

**Figure 11:**
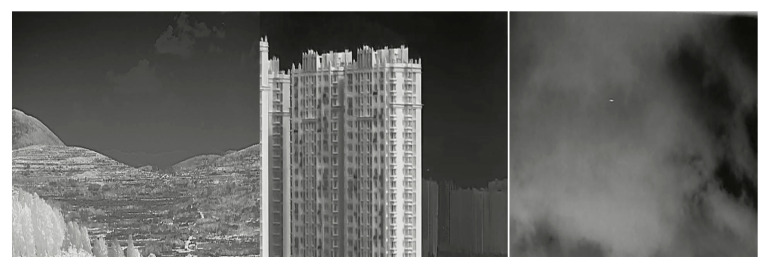
Example images from the Anti-UAV 410 data used for training and testing purposes.

**Figure 12:**
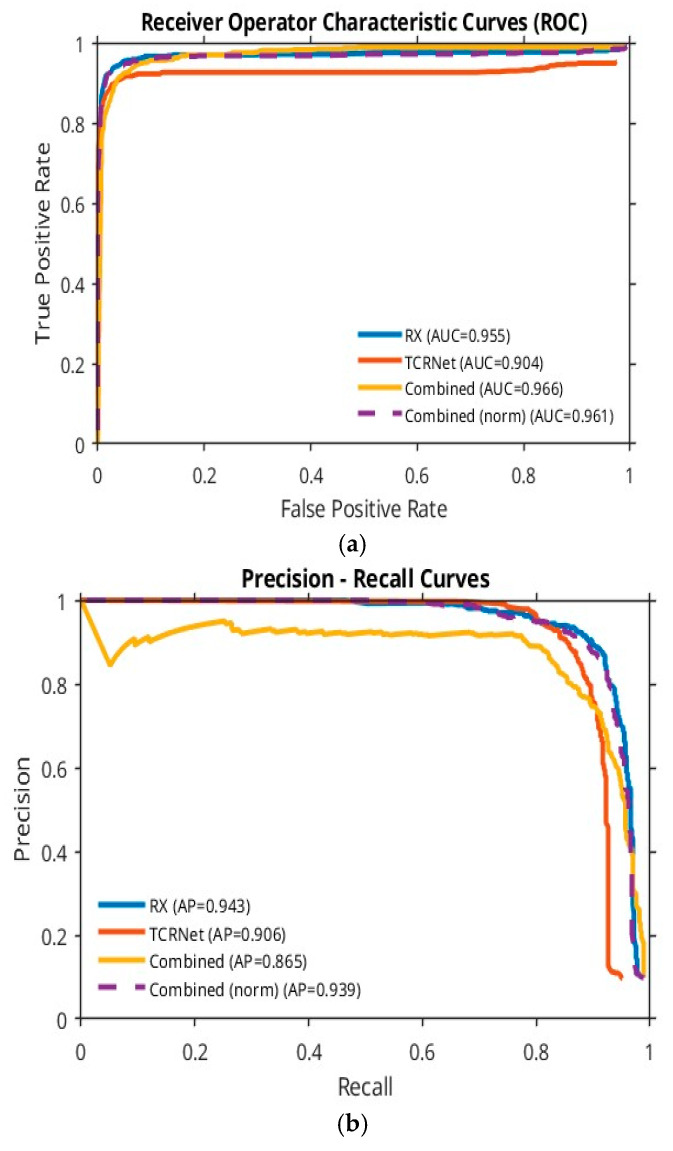
The ROC (**a**) and PR (**b**) curves generated using the Anti-UAV410 dataset demonstrate the generalization ability inherent to the TRX–TCRNet.

To further assess generalization beyond DAUB, we evaluated the model on the Anti-UAV410 dataset [32,33], a large MWIR benchmark encompassing varied illumination, seasons, and backgrounds. Some of the details of this dataset are given in Table 3, and sample images are shown in Figure 11. For consistency with the tiny-target nature of DAUB, we restricted evaluation to frames containing small UAV targets (1–10 pixels). ROC and PR curves (Figure 12) computed on this subset show that the performance of the algorithm remains comparable to its performance on the DAUB dataset shown in Figure 6. Thus, the TRX–TCRNet maintains stable detection behavior across different sensors and complex scenes, supporting its robustness under heterogeneous environmental conditions.

## 6. Conclusions

In this paper, we have introduced an algorithm for IR drone detection by processing spatio-temporal volumes in parallel using a temporal variant of the Reed Xiaoli (TRX) anomaly detector, and a light-weight CNN known as the Target to Clutter Ratio network (TCRNet) [2]. While the original RX detector was proposed for statistical anomaly detection in hyperspectral data, we employ it for detecting small drones as anomalous temporal changes in IR video streams. Specifically, the TRX detector is a constant false alarm rate (CFAR) algorithm that generates large per-pixel anomaly scores due to the movement of drones across the scene. The TCRNet is trained to discriminate between drones and clutter by learning the features that separate them in the spatio-temporal volume. For training the TCRNet, 3D spatio-temporal chips are extracted from the full frame videos, but during inference, the full volume is processed directly by the network. This leads to the TRX–TCRNet architecture which normalizes and additively combines the output of each algorithm to correctly localize targets while attenuating potential false alarms. This approach also ensures real-time efficiency by avoiding sequential dependencies, such as proposal generation and searching over many anchor boxes. By integrating temporal and spatial information with unprecedented efficiency, our method outperforms both deep single-frame detectors and handcrafted trackers. Our framework prioritizes efficiency over resource-heavy deep learning models and achieves nearly state-of-the-art performance on the DAUB dataset (97.40 mAP_50_) with only 0.83 M parameters and 0.17 GFLOPs. Ablation studies highlight the potential of adopting a local TRX detector with varying window sizes to further enhance detection accuracy. In comparison, other recently published methods require 8.7–17 more parameters along with much higher FLOPs (such as 145× more for ACM (24.66 G) [3], 769 times more for TRIDOS (130.72 G) [4], and 795 more for DNANet (135.24 G) [5]. This dramatic trade-off between task performance and computational cost makes the TRX–TCRNet uniquely suitable for embedded systems, mobile platforms, and resource-constrained environments where traditional deep learning approaches are impractical. This computational advantage positions our approach as a preferable solution for practical deployment scenarios where both high accuracy and resource efficiency are critical requirements. Future phases will expand evaluation to broader datasets, underscoring the pipeline’s potential for small-object detection and other tiny-object video applications across diverse hardware platforms.

## Figures and Tables

**Table 1:** Specifications of the DAUB dataset.

Annotations	Sequential	Classes	Target Size	Target Type	Number of Frames	Image Size	Scene Description
Bounding boxes	Yes	1	1~16 pixels	Drone	13,778	256 × 256	Ground background, forest, air background, plain, suburb, air round junction background

**Table 2:** Complexity comparison of inference. The best result is marked in bold.

Methods	Frames	mAP_50_	F1	FLOPs	Params	AUC
ACM [3]	1	64.02	79.86	24.66 G	3.04 M	64.2
TRIDOS [4]	5	**97.80**	**99.43**	130.72 G	14.13 M	97.8
DNANet [5]	1	89.93	95.29	135.24 G	7.22 M	90.3
RISTD [6]	1	81.05	90.26	76.28 G	3.28 M	81.4
SANet [7]	1	87.12	94.18	42.04 G	12.40 M	87.5
AGPCNet [8]	1	76.72	87.95	366.15 G	14.88 M	77.0
ISNet [9]	1	83.43	92.09	265.74 G	3.48 M	83.8
UIUNet [10]	1	86.41	93.23	456.70 G	53.06 M	86.7
RDIAN [11]	1	84.92	92.51	50.44 G	2.74 M	85.2
SSTNet [18]	5	95.59	98.09	123.59 G	11.95 M	96.1
TRX–TCRNet (Ours)	9	97.40	92.50	**0.17 G**	**0.83 M**	**98.8**

**Table 3:** Details of the Anti-UAV410 dataset.

Dataset	Annotations	Sequential	Target Size	Target Type	Number of Frames	Image Size	Scene Description
Anti-UAV410 [32]	Bounding boxes	√	1~10 pixels	Drone	17,071	512 × 640	Ground background, forest, air background, plain, suburb, mountain

## Data Availability

The original data presented in the study are openly available in https://doi.org/10.11922/csdata.2019.0074.zh (accessed on 29 December 2024).
